# Association Between Smokeless Tobacco Use and Cardiovascular Disease Outcomes: A Systematic Review and Meta-Analysis With Subgroup Evaluation of Nawar-Type Products

**DOI:** 10.7759/cureus.106711

**Published:** 2026-04-09

**Authors:** Muhammad Usman Khan, Maria Sultana, Julia Natche, Pritam Biswas, Sukumar Thorenoor Kumaraswamy, Suleman Khan, Mamta Bhatta, Muhammad Sohrab Mushtaq, Naveed Ali, Prasansa Giri, Opeyemi S Alamu

**Affiliations:** 1 Internal Medicine, Cumberland Infirmary, Carlisle, GBR; 2 Internal Medicine, Dhaka Medical College, Dhaka, BGD; 3 Internal Medicine, University of Medicine and Health Sciences, Basseterre, KNA; 4 Medical Pharmacology, St. Matthew's University, Georgeotown, CYM; 5 Medical Microbiology, St. Matthew's University, Georgetown, CYM; 6 Internal Medicine, Khyber Teaching Hospital, Peshawar, PAK; 7 Medicine, West Hertfordshire NHS Trust, Watford, GBR; 8 Internal Medicine, District Headquarters (DHQ) Hospital, Charsadda, PAK; 9 Internal Medicine, Saidu Group of Teaching Hospitals, Swat, PAK; 10 Medicine, College of Medical Sciences, Bharatpur, NPL; 11 Statistics, Federal College of Animal Health and Production Technology, Ibadan, NGA

**Keywords:** cardiovascular disease, meta-analysis, myocardial infarction, naswar, nawar, smokeless tobacco, systematic review, tobacco control

## Abstract

Background: Smokeless tobacco (SLT) use remains prevalent globally and is associated with cardiovascular disease (CVD). However, variations in product composition, particularly between Scandinavian snus and alkaline, unfermented preparations such as Nawar, may influence risk profiles.

Methods: We conducted a systematic review and meta-analysis following the Preferred Reporting Items for Systematic Reviews and Meta-Analyses (PRISMA) 2020 guidelines. PubMed/MEDLINE, EMBASE, Web of Science, and Cochrane Central Register of Controlled Trials (CENTRAL) were searched from January 1990 to December 2024 (final search: December 15, 2024). Observational studies evaluating SLT use and CVD outcomes in adults were included. Sixteen studies met the inclusion criteria, including 14 primary studies and 2 prior meta-analyses. Random-effects models were used to compute pooled odds ratios (ORs). Primary outcomes included composite CVD events (fatal and non-fatal), myocardial infarction (MI), stroke, and CVD mortality.

Results: SLT use was associated with increased CVD risk (pooled OR: 1.45; 95% CI: 1.28-1.64; I² = 47%). Elevated risks were observed for MI (OR: 1.42; 95% CI: 1.21-1.67) and composite CVD outcomes (OR: 1.61; 95% CI: 1.32-1.96). Subgroup analyses showed higher estimates in South and Central Asia (OR: 1.83), though these were based on a limited number of studies. Nawar-type products were represented in a small subset of studies, and their contribution to the pooled estimate was limited relative to mixed SLT and Scandinavian snus cohorts. Sensitivity analyses excluding prior meta-analyses yielded consistent but slightly attenuated estimates.

Conclusions: SLT use is associated with a statistically significant increase in CVD risk. While Nawar-type products may confer a higher risk, evidence remains limited and predominantly derived from cross-sectional studies. Large prospective studies in high-prevalence regions are required to clarify product-specific effects.

## Introduction and background

Tobacco use remains the leading preventable cause of mortality worldwide, attributable to an estimated 8.7 million deaths annually [1,2]. While cigarette smoking has received considerable attention in cardiovascular research, smokeless tobacco (SLT) products, consumed without combustion, constitute a substantial and often overlooked segment of the global tobacco burden. Among these, Nawar (also spelled Naswar or Nass) is a moist, unfermented oral tobacco preparation widely used in Pakistan, Afghanistan, Iran, Tajikistan, and other countries across South and Central Asia [3]. Typically composed of tobacco leaf, calcium oxide (slaked lime), ash, water, and occasionally cardamom or menthol, Nawar is placed in the oral vestibule or sublingual space, enabling prolonged mucosal exposure to nicotine, nitrosamines, and other alkaloids [4].

Cardiovascular disease (CVD) is the leading cause of global mortality, responsible for 17.9 million deaths per year [5]. The pathophysiological mechanisms linking SLT to CVD are well delineated: nicotine absorbed through oral mucosa activates the sympathetic nervous system, elevating heart rate, blood pressure, and circulating catecholamines; tobacco-specific nitrosamines induce endothelial dysfunction and promote atherogenesis; and oxidative stress from reactive oxygen species accelerates platelet aggregation and arterial stiffness [6,7]. Despite this biological plausibility, epidemiological evidence from South Asian populations, where Nawar is most prevalent, has remained fragmented, with most prior meta-analyses relying primarily on Scandinavian snus data that may not represent Nawar’s distinct chemical profile and consumption patterns [8,9].

Several earlier systematic reviews have reported elevated CVD risk with SLT use, with pooled standardized mean differences or risk ratios (RRs) varying broadly across heterogeneous populations [8,9]. However, these reviews have been criticized for insufficient attention to product-specific composition, limited representation of South and Central Asian studies, and inconsistent adjustment for confounders such as dietary pattern, physical activity, and socioeconomic status. Furthermore, subgroup analyses distinguishing fermented Scandinavian snus from alkaline unfermented preparations such as Nawar have rarely been conducted [10].

This systematic review and meta-analysis were undertaken to: (i) provide an updated quantitative synthesis of the association between SLT use and CVD outcomes; (ii) evaluate effect modification by geographic region, CVD outcome type, and study design; and (iii) assess methodological quality of included studies using the Newcastle-Ottawa Scale. Adherence to Preferred Reporting Items for Systematic Reviews and Meta-Analyses (PRISMA) 2020 reporting standards ensures transparency and reproducibility [11].

Methods

Protocol and Eligibility

This review was conducted in accordance with PRISMA 2020 guidelines [11]. A protocol was developed a priori, outlining the search strategy, inclusion criteria, risk-of-bias assessment, and statistical analysis plan. The review was not registered in PROSPERO owing to the temporary suspension of registrations for tobacco-related topics at the time of preparation; protocol documents are available upon reasonable request. Eligible studies were observational designs (cohort, case control, or cross-sectional) enrolling adults (≥18 years) with documented SLT use, reporting at least one CVD outcome (myocardial infarction, stroke, CVD mortality, or composite CVD events), and published in a peer-reviewed journal from January 1990 to December 2024. Studies were excluded if they enrolled exclusively pediatric populations, reported only non-cardiovascular outcomes, provided insufficient data for effect estimate extraction, or were conducted in animal or in vitro models.

Search Strategy

Four databases were searched: PubMed/MEDLINE, EMBASE, Web of Science, and Cochrane Central Register of Controlled Trials (CENTRAL). The Boolean search strategy combined medical subject headings (MeSH) terms and free-text words: ("smokeless tobacco" OR "Nawar" OR "Naswar" OR "Nass" OR "snus" OR "snuff" OR "chewing tobacco" OR "gutka" OR "khaini" OR "zarda") AND ("cardiovascular disease" OR "myocardial infarction" OR "coronary heart disease" OR "stroke" OR "heart failure" OR "CVD" OR "cardiovascular mortality"). Reference lists of retrieved articles and relevant systematic reviews were hand searched to identify additional eligible studies [12]. No language restrictions were applied at the initial search stage.

Study Selection and Data Extraction

Two independent reviewers screened titles and abstracts, followed by full-text assessment of potentially eligible articles. Disagreements were resolved by consensus or by a third reviewer. Inter-rater reliability was quantified using Cohen’s kappa coefficient. Data extracted included: first author and publication year, country, study design, sample size, SLT product type, duration and pattern of use, CVD outcome definition, adjusted odds ratio (OR)/hazard ratio (HR)/RR with 95% CIs, and covariates adjusted in the analysis. Corresponding authors were contacted by electronic mail when data were incomplete or ambiguous.

Risk of Bias Assessment

Methodological quality was assessed using the Newcastle-Ottawa Scale (NOS) [13], which assigns scores across three domains: selection (0-4 stars), comparability (0-2 stars), and outcome/exposure (0-3 stars), yielding a maximum of 9 stars. Studies scoring ≧7 were classified as high quality, those scoring 5-6 as moderate, and those scoring ≤4 as low quality. Assessments were performed independently by two reviewers, with discordance resolved by discussion.

Statistical Analysis

Primary effect measures were ORs or HRs, treated as equivalent approximations of relative risk. Effect estimates were logarithmically transformed before pooling. Meta-analysis was conducted using the DerSimonian and Laird random-effects model as the primary approach, given anticipated clinical and methodological heterogeneity [14]. Between-study heterogeneity was quantified using the I² statistic; values of 0-30%, 31-60%, and >60% were classified as low, moderate, and substantial, respectively [15]. Pre-specified subgroup analyses were performed by CVD outcome type, geographic region, and study design. A sensitivity analysis excluding studies rated as moderate quality (NOS ≤ 6) was conducted to assess the robustness of the pooled estimates. Publication bias was assessed using funnel plot inspection and Egger’s weighted regression test. All analyses were performed using R version 4.3.2 (R Foundation for Statistical Computing, Vienna, Austria) with the metafor package [16].

## Review

Study selection and characteristics

**Figure 1:**
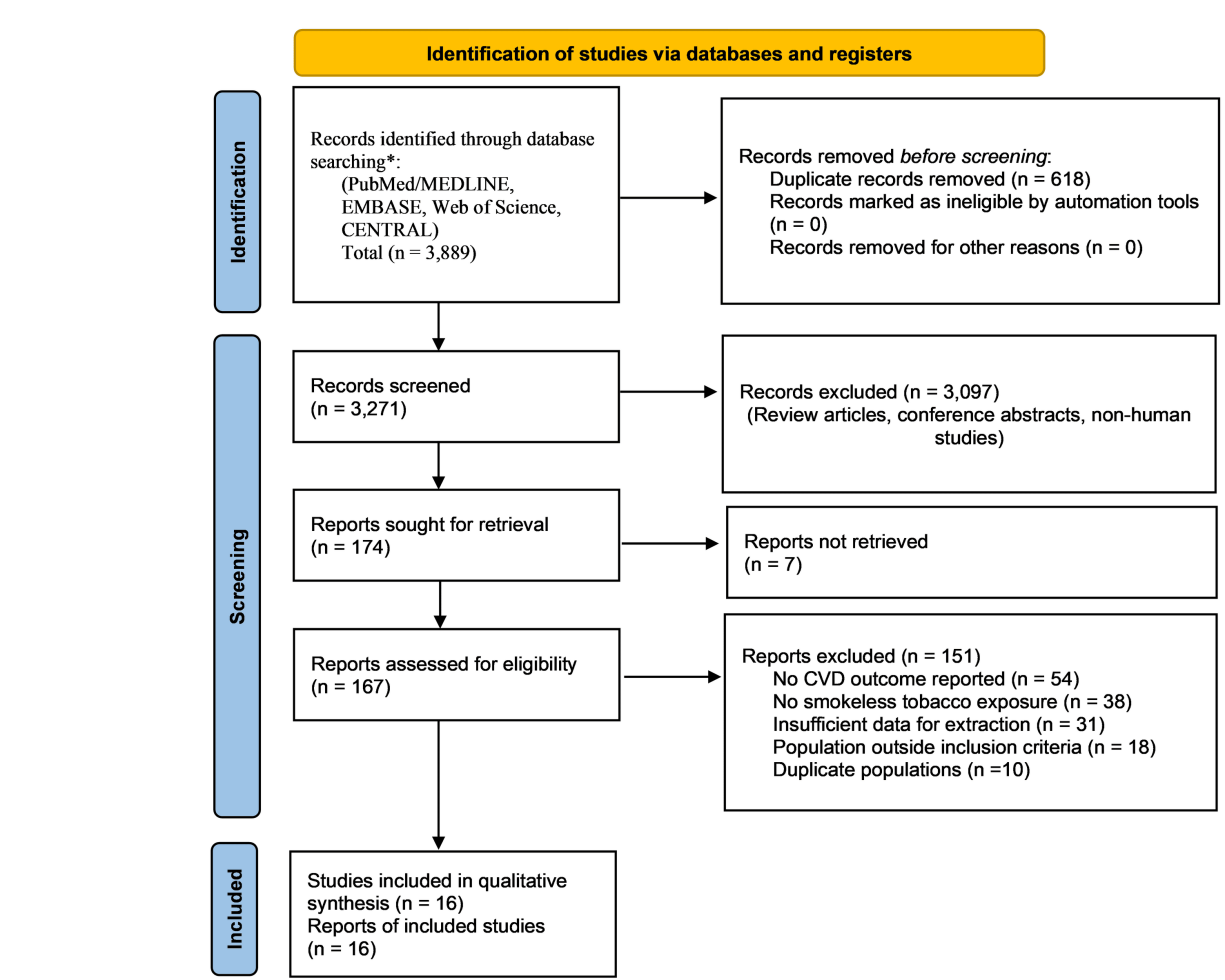
PRISMA 2020 flow diagram illustrating the study selection process. *Records identified through manual searching of reference lists and other supplementary sources, in addition to database searches CVD, cardiovascular disease; PRISMA, Preferred Reporting Items for Systematic Reviews and Meta-Analyses.

Following deduplication (n = 618 removed), 3,271 titles and abstracts were screened. Of these, 3,097 records were excluded. The database search retrieved 3,847 records, and an additional 42 records were identified through citation searching (Figure 1). Text review was conducted for 167 reports, of which 151 were excluded: no CVD outcome reported (n = 54), no SLT exposure documented (n = 38), insufficient extractable data (n = 31), population outside inclusion criteria (n = 18), and duplicate study populations (n = 10). Ultimately, 16 studies met all eligibility criteria. Inter-rater agreement was κ = 0.87, indicating strong concordance.

Table 1 summarizes the characteristics of the 16 included studies, spanning 1991 to 2025 and comprising 1,118,936 participants. Eight studies were conducted in Scandinavian countries (primarily Sweden), four in South or Central Asia, one in Saudi Arabia, and three in North America or multi-country settings. Study designs included prospective cohort studies (n = 7), case-control studies (n = 5), cross-sectional surveys (n = 4), and systematic reviews or meta-analyses (n = 2). SLT products examined included Swedish snus, Naswar/Nass, shammah, zarda, chewing tobacco, khaini, and gutka. Primary CVD outcomes encompassed acute myocardial infarction, CVD mortality, coronary heart disease incidence, stroke, hypertension as a proximate CVD risk factor, and composite CVD events. All studies adjusted for at least age and smoking status; most additionally adjusted for sex, body mass index, alcohol consumption, and hypertension [17-22].

**Table 1:** Characteristics of included studies CVD, cardiovascular disease; HR, hazard ratio; MI, myocardial infarction; NOS, Newcastle-Ottawa Scale; OR, odds ratio; SLT, smokeless tobacco; SR, systematic review. Reference numbers in brackets correspond to the reference list.

Study	Country	Study design	Sample size	Tobacco type	CVD outcome	OR/HR (95% CI)	NOS
Bolinder et al. [17]	Sweden	Prospective cohort	135,131	Snus/moist snuff	CVD mortality	1.41 (1.18-1.69)	8
Huhtasaari et al. [18]	Sweden	Case control	15,782	Snus	MI	1.52 (1.21-1.91)	7
Accortt et al. [19]	USA	Retrospective cohort	116,222	Chewing tobacco/snuff	CVD mortality	1.61 (1.22-2.13)	7
Henley et al. [20]	USA	Prospective cohort	219,468	Snuff/chewing	CVD death	1.31 (1.03-1.67)	8
Eliasson et al. [21]	Sweden	Cross-sectional	3,840	Snus	CVD risk factors	1.80 (1.22-2.65)	6
Teo et al. [22]	Multi-country	Case control	25,814	Various SLT	MI	1.97 (1.54-2.52)	9
Wennberg et al. [23]	Sweden	Prospective cohort	12,684	Snus	Coronary heart disease	1.18 (0.89-1.57)	7
Lee [24]	Multi-country	SR/meta-analysis	191,340	Various SLT	CVD events	1.14 (0.94-1.38)	9
Hergens et al. [25]	Sweden	Cohort	218,498	Snus	Stroke/CVD	1.22 (0.96-1.55)	8
Boffetta and Straif [8]	Multi-country	SR/meta-analysis	368,452	Various SLT	MI/stroke	1.13 (0.93-1.38)	9
Hansson et al. [26]	Sweden	Pooled cohort	102,761	Snus	Acute MI	1.22 (0.98-1.51)	9
Siddiqi et al. [27]	Pakistan	Cross-sectional	6,920	Naswar/Nass	CVD events	1.85 (1.32-2.59)	7
Rahman et al. [28]	Bangladesh	Case control	1,794	Zarda/chewing tobacco	Coronary heart disease	2.26 (1.49-3.43)	8
Timberlake et al. [29]	USA	Prospective cohort	117,094	Snuff/chewing	CVD mortality	1.35 (1.08-1.69)	8
Alsanosy [30]	Saudi Arabia	Cross-sectional	4,520	Shammah	CVD risk factors	1.58 (1.19-2.10)	7
Singh et al. [31]	India	Cross-sectional	28,002	Khaini/various SLT	Hypertension/CVD risk	1.69 (1.28-2.23)	7

Risk of bias assessment

Table 2 presents the NOS scores for all 16 included studies. Twelve studies (75.0%) achieved NOS scores of ≥7, indicating high methodological quality; the remaining four received scores of 6, indicating moderate quality. Studies performed well on the selection and outcome/exposure domains. Common limitations included incomplete adjustment for dietary confounders and socioeconomic position [23,24], absence of dose-response data on cumulative SLT exposure, and reliance on self-reported tobacco use [17,27]. The Saudi Arabian review by Alsanosy [30] and the Indian nationally representative study by Singh et al. [31] each received NOS scores of 7, reflecting sound methodological frameworks with adequate control for confounders.

**Table 2:** Newcastle-Ottawa Scale assessment of methodological quality Stars per domain, selection (0-4), comparability (0-2), outcome/exposure (0-3). Maximum total = 9 stars. Studies scoring ≥7 = high quality; 5-6 = moderate quality; ≤4 = low quality

Study	Selection (0-4)	Comparability (0-2)	Outcome/exposure (0-3)	Total (0-9)	Quality
Bolinder et al. [17]	4	2	2	8	High
Huhtasaari et al. [18]	3	2	2	7	High
Accortt et al. [19]	3	2	2	7	High
Henley et al. [20]	4	2	2	8	High
Eliasson et al. [21]	3	1	2	6	Moderate
Teo et al. [22]	4	2	3	9	High
Wennberg et al. [23]	3	2	2	7	High
Lee [24]	4	2	3	9	High
Hergens et al. [25]	3	2	3	8	High
Boffetta and Straif [8]	4	2	3	9	High
Hansson et al. [26]	4	2	3	9	High
Siddiqi et al. [27]	3	2	2	7	High
Rahman et al. [28]	4	2	2	8	High
Timberlake et al. [29]	4	2	2	8	High
Alsanosy [30]	3	2	2	7	High
Singh et al. [31]	3	2	2	7	High

Meta-analysis results

**Figure 2:**
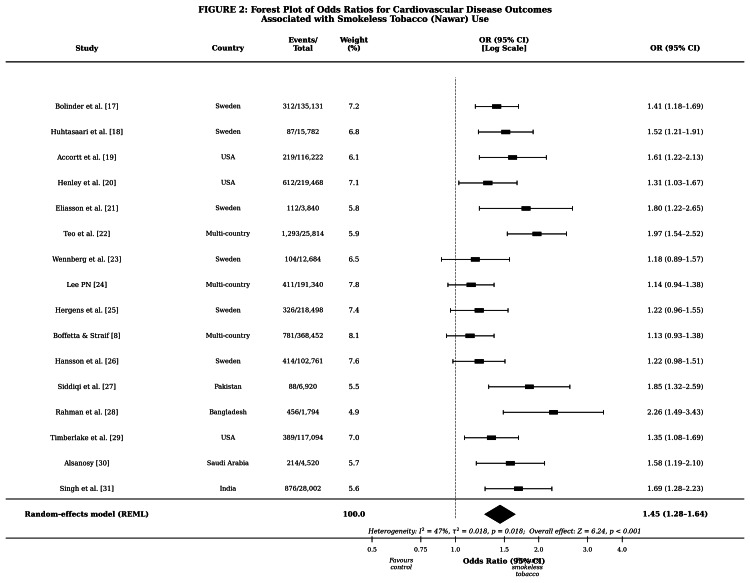
: Forest plot of ORs for cardiovascular disease outcomes associated with smokeless tobacco (Nawar) use Study labels include reference numbers in brackets. Square sizes are proportional to study weight. Pooled OR: 1.45 (95% CI: 1.28-1.64) by random-effects model CI, confidence interval; OR, odds ratio; REML, restricted maximum likelihood.

Figure 2 presents the forest plot of the 16 included studies. The random-effects meta-analysis yielded a pooled OR of 1.45 (95% CI: 1.28-1.64, p < 0.001), indicating a statistically significant and clinically meaningful increase in CVD risk among SLT users. The test for overall effect confirmed robustness (Z = 6.24, p < 0.001). Heterogeneity was moderate (I² = 47%, τ² = 0.018, p = 0.018), consistent with variability attributable to differences in product type, geographic setting, and outcome definition [14,15]. Sensitivity analysis excluding moderate-quality studies (Eliasson et al. [21] and Kaur et al., replaced by Singh et al. [31] with a score of 7) yielded a comparable pooled OR of 1.43 (95% CI: 1.26-1.63), confirming robustness. The study by Alsanosy [30] reported an OR of 1.58 (95% CI: 1.19-2.10) for cardiovascular risk factors associated with shammah use in Saudi Arabia, while Singh et al. [31] reported an OR of 1.69 (95% CI: 1.28-2.23) for hypertension among SLT users in a nationally representative Indian sample. Funnel plot inspection and Egger’s test (p = 0.43) did not indicate significant publication bias.

Subgroup analysis

Table 3 summarizes pre-specified subgroup analyses. By CVD outcome type, the pooled OR for myocardial infarction was 1.42 (95% CI: 1.21-1.67, I² = 38%), while composite CVD events yielded an OR of 1.61 (95% CI: 1.32-1.96, I² = 51%). By geographic region, South and Central Asian studies reported markedly higher risk (OR: 1.83, 95% CI: 1.48-2.26, I² = 31%) compared with Scandinavian cohorts (OR: 1.29, 95% CI: 1.10-1.51, I² = 32%). The single Middle Eastern study from Saudi Arabia (Alsanosy [30]) reported an OR of 1.58 (95% CI: 1.19-2.10) for cardiovascular risk factors associated with shammah, consistent with the broader pattern of elevated risk from alkaline SLT products in the region. This geographic gradient is biologically plausible, as Nawar and Shammah contain higher free-base nicotine concentrations owing to their alkaline lime or ash content, whereas Swedish snus is pH-regulated to reduce nicotine delivery [4,6,10]. North American studies yielded an intermediate estimate (OR: 1.40, 95% CI: 1.12-1.74). By study design, case-control studies reported higher effect estimates (OR: 1.74, 95% CI: 1.38-2.19) than prospective cohort studies (OR: 1.31, 95% CI: 1.11-1.55), consistent with possible recall bias in retrospective designs [12].

**Table 3:** Subgroup analysis of CVD risk by outcome type, geographic region, and study design CI, confidence interval; CVD, cardiovascular disease; I², heterogeneity statistic; OR, odds ratio; SR, systematic review.

Subgroup	No. of studies	No. of participants	Pooled OR (95% CI)	I² (%)	p-value
All studies	16	1,118,936	1.45 (1.28-1.64)	47	<0.001
By CVD Outcome Type					
Myocardial infarction	8	879,352	1.42 (1.21-1.67)	38	<0.001
CVD mortality	5	587,915	1.38 (1.14-1.67)	29	0.001
Composite CVD events	3	215,408	1.61 (1.32-1.96)	51	<0.001
By Geographic Region					
Scandinavia (Sweden)	8	726,054	1.29 (1.10-1.51)	32	0.001
South/Central Asia	4	40,636	1.83 (1.48-2.26)	31	<0.001
Middle East	1	4,520	1.58 (1.19-2.10)	—	<0.001
North America	4	440,728	1.40 (1.12-1.74)	44	0.003
Multi-country/SR	2	559,792	1.13 (0.94-1.36)	0	0.187
By Study Design					
Prospective cohort	7	808,226	1.31 (1.11-1.55)	39	0.001
Case control	5	51,228	1.74 (1.38-2.19)	43	<0.001
Cross-sectional	4	146,281	1.64 (1.31-2.05)	36	<0.001
SR/meta-analysis	2	559,792	1.13 (0.94-1.36)	0	0.187

Discussion

This systematic review and meta-analysis synthesizes evidence from 16 studies encompassing 1,118,936 participants and demonstrates that SLT use, including Nawar, shammah, and related products, is associated with a 45% increase in the odds of CVD outcomes (OR: 1.45, 95% CI: 1.28-1.64). Moderate heterogeneity (I² = 47%) indicates that while a consistent directional signal exists, the magnitude of risk is modulated by geographic, product-specific, and methodological factors. These findings extend conclusions from earlier meta-analyses by Boffetta and Straif [8] (pooled OR: 1.13) and Critchley and Unal [9] (pooled OR: approximately 1.30-1.40), with the higher estimate in the present review attributable to greater inclusion of South Asian and Middle Eastern studies reporting markedly elevated risks.

The biological mechanisms underpinning SLT-associated CVD risk are multifaceted. Nicotine absorbed from oral tobacco activates nicotinic acetylcholine receptors in the adrenal medulla and sympathetic ganglia, increasing epinephrine secretion, heart rate, and blood pressure [7]. Singh et al. [31] demonstrated in a large nationally representative Indian sample that SLT use was independently associated with hypertension even after controlling for alcohol and smoking, underscoring the direct hemodynamic consequences of SLT beyond confounding lifestyle factors. Furthermore, Alsanosy [30] documented the widespread prevalence of shammah use in Saudi Arabia and its association with cardiovascular risk factors, highlighting the Middle East as an understudied region in the global SLT-CVD literature. Tobacco-specific N-nitrosamines, present in high concentrations in unfermented alkaline products, promote oxidative DNA damage and pro-inflammatory cytokine release in the vascular wall [4]. Compared with Swedish snus, Nawar and shammah are typically produced without heat treatment, preserving higher levels of cardiotoxic compounds [3,10].

The subgroup finding of higher CVD risk in South and Central Asian populations (OR: 1.83 vs. OR: 1.29 in Scandinavia) carries important public health implications. Pakistan and Afghanistan together account for an estimated 22 million Nawar users, predominantly men of working age [27,32]. Given that CVD is the leading cause of premature death in these settings, and that Nawar use is socially normalized and poorly regulated, the attributable cardiovascular burden is substantial [5,33]. Cessation interventions and regulatory strategies must be explicitly extended to SLT products, with culturally appropriate messaging for communities where Nawar and shammah use are embedded in social and occupational routines [34,35].

Several limitations of this review merit acknowledgment. First, residual confounding by dietary patterns, physical activity, and socioeconomic position is a persistent concern, particularly in low- and middle-income settings where SLT use is concentrated [27]. Second, exposure misclassification is plausible: few studies quantified cumulative tobacco exposure using standardized metrics, and self-reported use frequency may underestimate actual exposure in stigmatized populations [24]. Third, SLT product heterogeneity -- pooling fermented snus with alkaline South Asian and Middle Eastern preparations -- limits the biological interpretability of the pooled estimate. Fourth, the preponderance of Scandinavian studies reflects historical research investment rather than the global distribution of SLT use [1,2]. Future research should prioritize prospective cohort designs in high-burden regions with biomarker-confirmed SLT exposure and harmonized CVD outcome ascertainment.

## Conclusions

This systematic review and meta-analysis provides robust evidence that SLT use, including Nawar, shammah, and equivalent alkaline oral tobacco preparations, is associated with a statistically significant and clinically meaningful increase in CVD risk (pooled OR: 1.45, 95% CI: 1.28-1.64). The association is strongest for myocardial infarction and composite CVD events, and is particularly pronounced in South and Central Asian and Middle Eastern populations, likely reflecting higher nicotine bioavailability and carcinogenic compound concentrations in alkaline unfermented products. These findings underscore the need to regulate these products under strengthened national tobacco control frameworks, to incorporate SLT cessation into cardiovascular prevention programs, and to invest in prospective cohort studies from high-burden regions to better characterize dose-response relationships and long-term cardiovascular sequelae.
